# Preparing students for psychiatry OSCE's in the COVID-19 pandemic. How can PsychSocs help?

**DOI:** 10.1192/bjo.2021.101

**Published:** 2021-06-18

**Authors:** Isabella Conti, Chloe Gilkinson

**Affiliations:** Queen's University Belfast

## Abstract

**Aims:**

The need for social distancing during the COVID-19 pandemic has led medical schools to make use of video conferencing platforms in their Objective Structed Clinical Exams (OSCE) for the first time. Additionally the suspension of OSCE's in 2020 due to the pandemic ,has meant this cohort of final year students have never been examined on psychiatry skills. Our aims were to assess if our student psychiatry society (PsychSoc) run OSCE could help to prepare medical students for novel virtual stations ahead of their final examinations, and how this format of mock examination could be improved in the future.

**Method:**

Our PsychSoc (QUB Mind Matters) hosted a virtual mock psychiatry OSCE for 24 final year medical students using the video conference platform Zoom, approximately 1 month before their finals. The OSCE comprised 4 stations each lasting 8 minutes, and covered psychiatric history taking, risk assessment and drug counselling. Stations were marked by psychiatry trainees in individual breakout rooms to closely simulate real examination conditions. A post-event online questionnaire was distributed to all participants. 5-point Likert scales and free text responses were used to gather feedback regarding the content and delivery of the mock. A response rate of 100% was achieved (n = 24).

**Result:**

The feedback from students was overwhelmingly positive, with 100% (n = 24) agreeing/strongly agreeing that the mock OSCE left them feeling more prepared for their final exam. 95.8% (n = 23) agreed that the opportunity to practice virtual OSCE stations improved their confidence, and all 24 students agreed/strongly agreed that they would like more practice of virtual OSCE stations. A common theme that emerged when asked how our mock could have been improved was the need for a group feedback session covering common pitfalls in addition to individual feedback.

**Conclusion:**

The lack of clinical experience and shift towards online learning has led to increased stress around clinical exams in the student population. PsychSocs can supplement formal teaching by providing students the opportunity to practice virtual communication and history taking skills that are not always covered in their undergraduate curriculum. However, as a psychiatry society our mock only assessed psychiatry skills, many of which may be relatively well suited to an online format. We would welcome further evaluation of the applicability of student run virtual mock OSCE's to other specialties. We have demonstrated that PsychSocs can offer much needed practice for students through mock OSCE's and have highlighted ways to enhance their delivery.

